# Phase entropy of gated SPECT-MPI for predicting major adverse cardiovascular events: incremental prognostic value beyond perfusion and function

**DOI:** 10.3389/fcvm.2026.1839622

**Published:** 2026-07-07

**Authors:** Yuxin Xiao, Yuting Zhao, Zhifang Wu, Xiaoli Zhang, Yuetao Wang, Minfu Yang, Sijin Li

**Affiliations:** 1 Department of Nuclear Medicine, First Hospital of Shanxi Medical University, Shanxi Medical University, Taiyuan, Shanxi, China; 2 Shanxi Key Laboratory of Molecular Imaging, Shanxi Medical University, Taiyuan, Shanxi, China; 3 Collaborative Innovation Center for Molecular Imaging of Precision Medicine Shanxi Medical University, Taiyuan, Shanxi, China; 4 Laboratory for Molecular Imaging, Department of Nuclear Medicine, Beijing Anzhen Hospital, Capital Medical University, Beijing, China; 5 Department of Nuclear Medicine, the Third Affiliated Hospital of Soochow University, Changzhou, China; 6 Institute of Clinical Translation of Nuclear Medicine and Molecular Imaging, Soochow University, Changzhou, China; 7 Department of Nuclear Medicine, Changzhou Clinical Medical Center, Changzhou, China; 8 Department of Nuclear Medicine, Beijing Chao-Yang Hospital, Capital Medical University, Beijing, China

**Keywords:** major adverse cardiovascular events, myocardial perfusion imaging, phase analysis, phase entropy, total perfusion deficit

## Abstract

**Background:**

Gated SPECT myocardial perfusion imaging (MPI) phase analysis provides a quantitative assessment of left ventricular mechanical synchrony, enabling cardiovascular risk stratification. Phase entropy, a derived imaging biomarker, reflects the spatiotemporal heterogeneity of myocardial contraction. However, its prognostic significance and clinically applicable threshold values have not been rigorously validated. This study aimed to determine the independent prognostic value of phase entropy for predicting major adverse cardiovascular events (MACE).

**Results:**

Among 1,674 coronary artery disease patients (median age 62.0 years, 66.2% male) followed for a median 36.6 months, an U-shaped association between phase entropy and major adverse cardiovascular events was identified (*P* = 0.005). Patients with abnormal phase entropy (>53% or ≤31%) exhibited significantly lower survival than those with normal values (31%–53%, *P* < 0.001). After multivariable adjustment, phase entropy remained an independent major adverse cardiovascular events predictor, and its addition to a model including total perfusion deficit (TPD) and left ventricular ejection fraction (LVEF) improved major adverse cardiovascular events prediction (*P* *<* 0.05).

**Conclusion:**

Phase entropy ≤31% or >53% correlates with reduced survival. This biomarker independently predicts major adverse cardiovascular events and provides incremental prognostic value beyond TPD and LVEF, with ≤31% identifying a high-risk subgroup that merits intensified clinical monitoring.

## Introduction

Phase analysis of gated SPECT-MPI quantifies left ventricular mechanical dyssynchrony (LVMD), enabling integrated assessment of cardiac mechanical function and refined cardiovascular risk stratification (1). As a biomarker derived from this technique, phase entropy quantifies global mechanical heterogeneity and independently predicts major adverse cardiovascular events, including myocardial infarction (MI), clinically significant arrhythmias, and heart failure (HF) (2–4).

As a quantitative measure of LVMD derived from phase analysis, phase entropy directly correlates with the degree of spatiotemporal contraction heterogeneity, manifested as abnormal temporal dispersion of regional myocardial contraction (5). LVMD is an established predictor of adverse outcomes in heart failure populations and candidates for cardiac resynchronization therapy (CRT) (6, 7). Phase entropy provides sensitive quantification of LVMD severity, thereby facilitating refined risk stratification in these patient cohorts.

Quantitative phase analysis of gated SPECT-MPI demonstrated strong concordance with tissue Doppler-derived LVMD measurements (8). Notably, elevation in phase entropy precedes detectable abnormalities in conventional echocardiographic indices such as LVEF, supporting its utility for early cardiovascular risk stratification (9). Beyond conventional perfusion and functional parameters, stress phase entropy enhances detection of subclinical myocardial ischemia in patients with CAD by quantifying stress-induced mechanical heterogeneity. This early biomarker signal facilitates proactive risk stratification for incident major adverse cardiovascular events (10).

Current evidence specifically addressing phase entropy remains limited, with existing clinical insights primarily extrapolated from pathophysiology related mechanical indices or constrained by small sample sizes (11, 12). This study aims to establish the independent association between phase entropy thresholds and major adverse cardiovascular events after adjustment for established risk factors and quantify its incremental prognostic value beyond conventional predictors including TPD and LVEF using validated net reclassification improvement (NRI) and integrated discrimination improvement (IDI) metrics.

## Methods

### Study population

We retrospectively analyzed 1,858 consecutive patients undergoing resting gated SPECT-MPI at the First Hospital of Shanxi Medical University (January 2016 to March 2021). Exclusion criteria comprised: (1) coronary revascularization within 90 days post-imaging (13) (*n* = 63); (2) severe arrhythmias (*n* = 21); (3) ventricular paced rhythm (*n* = 6); (4) prior non-coronary cardiac surgery (*n* = 41); (5) incomplete imaging data (*n* = 53). The final cohort included 1,674 patients. The exclusion of patients who underwent early revascularization was necessitated by the potential for SPECT-MPI results to confound the assessment of cardiovascular outcomes by influencing revascularization decisions (13). This study complied with the Declaration of Helsinki and received approval from the Institutional Review Board of the First Hospital of Shanxi Medical University (ID: 2022-K-128). Written informed consent was obtained from all participants.

### Clinical data

Collected clinical data comprised demographic characteristics: age, sex, body mass index (BMI); cardiovascular risk factors: smoking, diabetes mellitus, hypertension, dyslipidemia, family history of premature CAD (first-degree relative <55 years); prior MI, cerebrovascular events (hemorrhagic/ischemic stroke), peripheral vascular disease (PVD); revascularization history: percutaneous coronary intervention (PCI), coronary artery bypass grafting (CABG); electrocardiographic abnormalities: ST-segment deviation (elevation/depression ≥0.1 mV), T-wave inversion ≥2 contiguous leads, pathological Q waves, new-onset bundle branch block, sustained ventricular tachycardia (>30 seconds) (14); medication usage. Coronary stenosis severity and the number of affected major epicardial vessels were quantified using coronary angiography (CAG) and/or coronary computed tomography angiography (CCTA), with significant stenosis defined as ≥50% luminal narrowing in arteries ≥2.0 mm in diameter (15).

### SPECT-MPI protocol

All patients underwent resting gated SPECT-MPI using an IQ-SPECT dual-detector system (Symbia T16, Siemens Healthineers, Germany). After ≥4 hours of fasting, intravenous injection of Tc-99 m sestamibi (740–925 MBq) was administered. Patients consumed a high-fat-content meal 15–20 minutes prior to imaging to enhance hepatobiliary clearance. Electrocardiographic gating electrodes were positioned on the chest. Patients were positioned supine with arms elevated in dedicated immobilization devices. Image acquisition commenced 60 minutes post-injection, lasting 8 minutes with the following parameters Matrix: 128 × 128; Acquisition mode: Step-and-shoot; Projections: 34 frames over 208° orbit; Time per frame: 25 seconds. Reconstruction was performed using ordered subset expectation maximization with attenuation correction. Left ventricular contours were automatically generated using quantitative gated SPECT (QGS) software.

### Image analysis

GMPI images must be analyzed by at least two experienced nuclear medicine physicians. Any discrepancies in interpretation should be resolved by a third senior physician. Quantitative analysis was performed using QGS and quantitative perfusion SPECT (QPS) software (Cedars-Sinai Medical Center, Los Angeles, CA) (16), generating the following standardized metrics perfusion parameters: summed stress score, summed rest score. Functional Parameters: LVEF; End-diastolic volume; End-systolic volume. Mechanical Synchrony Indices: Bandwidth; Phase standard deviation (SD); Phase entropy. Phase Analysis Metrics: Histogram bandwidth, peak phase.

### Phase analysis methodology

Phase analysis involves reconstructing gated MPI into 16-frame cine sequences. Time-activity curves derived from myocardial samples undergo first-harmonic Fourier transformation to compute two key parameters: Phase angle; Amplitude. This generates three functional representations: Phase distribution map; Amplitude map; Phase cine display. The phase histogram quantifies the frequency distribution of myocardial contraction onset times: *X*-axis, Phase angles (0°–360°), where 0° = R-wave peak (electrocardiographic gating reference), 360° = Subsequent R-wave (completing one cardiac cycle); *Y*-axis, Relative frequency of voxels (%). LVMD severity is quantified by histogram bandwidth (range containing 95% of voxels) and phase SD distribution (17). Third-harmonic Fourier decomposition of gated SPECT data provides specific quantification of diastolic dyssynchrony (18), complementing conventional systolic dyssynchrony assessment in comprehensive LVMD evaluation.

Phase histogram derived parameters including bandwidth, phase SD and entropy were quantified using validated algorithms in QGS software (17). Phase entropy quantification in gated SPECT-MPI applies Shannon's information entropy theory to myocardial phase analysis. Using the equation (*H* = −∑*_i_ p_i_* log *p_i_*_,_ where *p_i_* is the probability of state *i*). Entropy values (range: 0–1) increase with greater phase dispersion, reflecting mechanical dyssynchrony severity. Normalization by log(*n*) confines *H* to the interval [0,1], where 0 indicates perfect synchrony and 1 indicates maximal asynchrony (achieved when states are uniformly distributed). Clinically reported entropy values are scaled to percentage (0%–100%) by multiplying by 100 (19).

### Study endpoints

The primary endpoint was a composite major adverse cardiovascular events, comprising: All-cause death (20); Nonfatal MI; Late coronary revascularization (>90 days post-MPI) including PCI or CABG; Readmission for HF or unstable angina. If multiple major adverse cardiovascular events occur simultaneously, record the most serious event. Secondary endpoints were defined as “hard events”, which included all-cause death, nonfatal MI, and late revascularization.

All follow-ups were conducted between April and August 2022. Data collection utilized multimodal methods including electronic health records (EHR) review and structured telephone interviews with patients or proxies. Follow-up duration was calculated from the index SPECT-MPI date until the first occurrence of major adverse cardiovascular events.

### Statistical analysis

Categorical variables are presented as frequencies (%). Continuous variables are summarized as medians with interquartile ranges (IQRs). Group comparisons employed: *χ^2^* test (or Fisher's exact test for expected cell counts <5) for categorical variables; Independent samples t-test (normally distributed data) or Mann–Whitney U test (non-normal data) for continuous variables, with normality assessed by Shapiro–Wilk test (*α* = 0.05). Annualized major adverse cardiovascular events incidence rates (events per 100 person-years) were calculated across entropy bandwidth and phase SD deciles. Optimal prognostic thresholds for phase parameters (entropy, bandwidth, phase SD) were derived from receiver operating characteristic curves using Youden's index maximization criterion (21). Kaplan–Meier survival curves were generated to evaluate major adverse cardiovascular events-free survival, stratified by TPD, LVEF, and phase variables. The curves were compared using log-rank tests, with Bonferroni correction applied for multiple comparisons.

Associations between phase parameter quartiles and major adverse cardiovascular events were assessed using Cox proportional hazards regression. For continuous phase variables, we employed dual parameterization approaches: Linear continuous scaling; Restricted cubic splines (RCS) with 3 knots. Results are reported as hazard ratio (HR) with 95% confidence interval (CI). Multivariable models included covariates significant at *P* *<* 0.01 in univariate analyses: Clinical factors included age, sex, BMI, hypertension, dyslipidemia, diabetes, family history of CAD, smoking status, PVD, previous MI and prior PCI/CABG.

Global chi-square analyses were performed using Cox models and likelihood ratio tests to evaluate the incremental value of LVEF relative to TPD alone, as well as the incremental value of phase variables relative to the combination of LVEF and TPD.TPD ≥ 5% (13) was defined as abnormal and LVEF < 50% (22) was defined as abnormal. All analyses used R 4.5.1 (R Foundation). Two-sided *P* < 0.05 defined statistical significance.

## Results

### Study population and clinical data

Table 1 presents baseline characteristics of the overall cohort and subgroups stratified by major adverse cardiovascular events occurrence during follow-up. Compared to the non-major adverse cardiovascular events group, patients experiencing major adverse cardiovascular events were significantly older (median 64.0 vs. 60.0 years; *P* < 0.001). Males constituted 69.2% of the major adverse cardiovascular events group compared to 64.2% in the non-major adverse cardiovascular events group (*P* = 0.036). Comorbidities including hypertension, dyslipidemia, diabetes, PVD, prior MI, and prior revascularization (PCI/CABG) were more prevalent in the major adverse cardiovascular events group (*P* < 0.05). During a median follow-up of 36.6 months (IQR 20.5–58.7), 662 adjudicated major adverse cardiovascular events events occurred. The secondary endpoint events were observed in 290 patients, including 71 deaths, 7 nonfatal MI, and 212 cases of late revascularization.

**Table 1:** Baseline characteristics in the overall population.

Number	Overall n=1674	No MACE n=1012	MACE n=662	*P*-value
Age (years)	62.0 [53.0, 71.0]	60.0 [51.8, 68.0]	64.0 [55.0, 73.8]	<0.001
Male	1108 (66.2%)	650 (64.2%)	458 (69.2%)	0.036
BMI (kg/m^2^)	24.3 [22.2, 26.7]	24.5 [22.2, 26.8]	24.2 [22.2, 26.3]	0.146
Hypertension	956 (57.1%)	532 (52.6%)	424 (64.0%)	<0.001
Dyslipidemia	625 (37.3%)	346 (34.2%)	279 (42.1%)	0.003
Diabetes Mellitus	437 (26.1%)	225 (22.2%)	212 (32.0%)	<0.001
Family History of CAD	540 (32.3%)	320 (31.6%)	220 (33.2%)	0.493
Smoking	728 (43.5%)	423 (41.8%)	305 (46.1%)	0.085
PVD	887 (53.0%)	527 (52.1%)	360 (54.4%)	0.003
History of MI	565 (33.8%)	253 (25.0%)	312 (47.1%)	<0.001
History of CAD	1489 (88.9%)	860 (85.0%)	629 (95.0%)	<0.001
History of PCI	113 (6.8%)	49 (4.8%)	64 (9.7%)	<0.001
History of CABG	99 (5.9%)	42 (4.2%)	57 (8.6%)	<0.001

The values are expressed as the median [25th and 75th percentiles] or the number of patients (%). BMI, body mass index; CAD, coronary artery disease; CABG, coronary artery bypass graft surgery; MI, myocardial infarction; PCI, percutaneous coronary intervention; PVD, peripheral vascular disease; MACE, major adverse cardiac events.

### Correlations between spect variables and major adverse cardiovascular events

**Figure 1:**
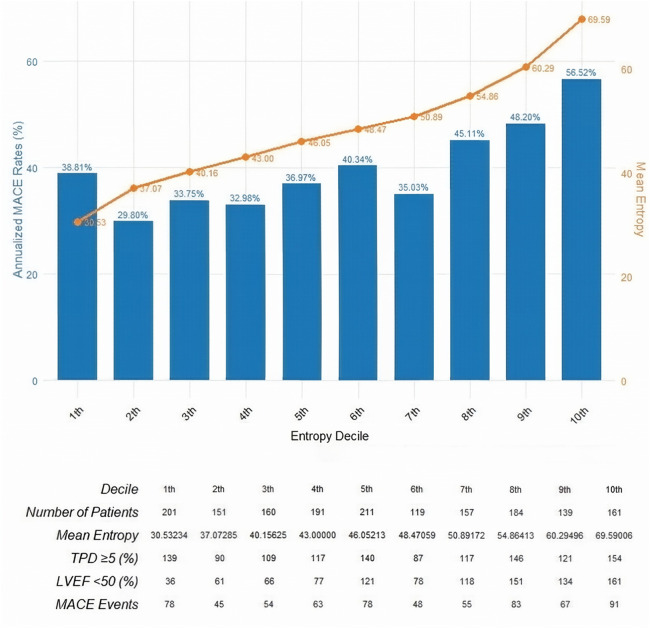
Annualized incidence of major adverse cardiovascular events for entropy. The bar chart illustrates the annualized major adverse cardiovascular events incidence rates (%) across ten deciles of phase entropy (*n* = 1,674 total patients; *n* = 119–211 per decile). The orange line represents the mean entropy value for each decile. Annualized major adverse cardiovascular events rates were calculated as events per 100 person-years. Statistical comparisons across deciles were performed using the *χ*^2^ test for categorical variables. MACE, major adverse cardiovascular events; LVEF, left ventricular ejection fraction; TPD, total perfusion deficit.

The gated SPECT results are shown in Table 2. Patients with major adverse cardiovascular events have higher entropy bandwidth and TPD. The LVEF of major adverse cardiovascular events patients is relatively low. Figure 1 shows the event rate of the deciles of phase entropy. With the increase of entropy, the annualized major adverse cardiovascular events rate increases and higher prevalence of abnormal myocardial perfusion (TPD ≥ 5%) and systolic dysfunction (LVEF < 50%) (Figure 1). As the deciles of bandwidth and phase SD increase, the annual major adverse cardiovascular events rate also increases (Supplementary Figures S1, S2).

**Table 2:** Imaging characteristics.

Number	Overall n=1674	No MACE n=1012	MACE n=662	*P*-value
Entropy (%)	47.0 [40.0, 54.0]	46.0 [39.0, 52.0]	48.0 [41.0, 57.0]	<0.001
Phase SD (°)	18.9 [11.7, 27.3]	18.2 [11.5, 26.7]	19.9 [12.2, 28.3]	0.062
Bandwidth (°)	54.0 [36.0, 84.0]	54.0 [36.0, 78.0]	54.0 [42.0, 90.0]	0.001
TPD (%)	8.0 [4.0, 16.0]	7.0 [4.0, 13.0]	10.0 [5.0, 22.0]	<0.001
LVEF (%)	47.0 [36.0, 54.0]	48.0 [39.0, 55.0]	44.5 [32.0, 53.0]	<0.001

The values are expressed as the median [25th and 75th percentiles]. LVEF, left ventricular ejection fraction; SD, standard deviation; TPD, total perfusion deficit; MACE, major adverse cardiac events.

### Baseline characteristics stratified by phase entropy

Baseline clinical characteristics and SPECT parameters of the 1674 enrolled patients stratified by phase entropy are presented in Table 3. Overall, 1142 patients (68.2%) were categorized into the intermediate phase entropy group (31–53), 441 (26.3%) into the high phase entropy group (>53), and 91 (5.4%) into the low phase entropy group (≤31). Age and BMI were comparable across the three groups. No significant differences were also observed in hypertension, family history of CAD, and prior history of PCI or CABG. By contrast, male sex, dyslipidemia, current smoking and prior MI were significantly more prevalent in patients with high phase entropy (all *P* < 0.001). Notably, SPECT imaging indices reflecting myocardial mechanical synchrony, perfusion and cardiac function differed markedly among groups. The high phase entropy group exhibited elevated phase SD, bandwidth and TPD, accompanied by a substantially reduced LVEF (all *P* < 0.001).

**Table 3:** Baseline clinical and SPECT-MPI characteristics of patients stratified by phase entropy level.

Number	Overall n=1674	Entropy31-53 n=1142	Entropy > 53 n=441	Entropy≤31 n=91	*P*-value
Age (years)	62.0 [53.0, 71.0]	61.0 [53.0, 70.0]	62.0 [54.0, 71.0]	62.0 [57.0, 72.0]	0.428
Male	1108 (66.2%)	731 (64%)	332 (75.3%)	45 (49.5%)	<0.001
BMI (kg/m^2^)	24.3 [22.2, 26.7]	24.5 [22.2, 26.8]	24.2 [22.2, 26.7]	23.5 [22.4, 25.6]	0.069
Hypertension	956 (57.1%)	640 (56%)	266 (60.3%)	50 (54.9%)	0.278
Dyslipidemia	625 (37.3%)	312 (27.3%)	266 (60.3%)	47 (51.6%)	<0.001
Diabetes Mellitus	437 (26.1%)	282 (24.7%)	124 (28.1%)	31 (34.1%)	0.078
Family History of CAD	540 (32.3%)	376 (32.9%)	137 (31.1%)	27 (29.7%)	0.671
Smoking	728 (43.5%)	473 (41.4%)	229 (51.9%)	26 (28.6%)	<0.001
PVD	887 (53.0%)	629 (55.1%)	206 (46.7%)	52 (57.1%)	<0.001
History of MI	565 (33.8%)	312 (27.3%)	231 (52.4%)	22 (24.2%)	<0.001
History of CAD	1489 (88.9%)	994 (87.0%)	416 (94.3%)	79 (86.8%)	<0.001
History of PCI	113（6.8%）	77 (6.7%)	31 (7.0%)	5 (5.5%)	0.88
History of CABG	99 (5.9%)	68 (6.0%)	27 (6.1%)	4 (4.4%)	0.79
Phase SD (°)	18.9 [11.7, 27.3]	16.0 [10.8 23.9]	27.5 [21.9, 37.9]	5.5 [4.5, 10.1]	<0.001
Bandwidth (°)	54.0 [36.0, 84.0]	48.0 [36.0, 60.0]	102.0 [78.0, 144.0]	24.0 [18.0, 24.0]	<0.001
TPD (%)	8.0 [4.0, 16.0]	6.0 [4.0, 11.0]	22.0 [9.0, 36.0]	6.0 [4.0, 9.0]	<0.001
LVEF（%）	47.0 [36.0, 54.0]	49.0 [43.0, 56.0]	29.0 [21.0, 38.0]	60.0 [54.5. 69.0]	<0.001

The values are expressed as the median [25th and 75th percentiles] or the number of patients (%). BMI, body mass index; CAD, coronary artery disease; CABG, coronary artery bypass graft surgery; MI, myocardial infarction; PCI, percutaneous coronary intervention; PVD, peripheral vascular disease; MACE, major adverse cardiac events; LVEF, left ventricular ejection fraction; SD, standard deviation; TPD, total perfusion deficit.

### Relationships between phase analysis and other MPI variables

**Figure 2:**
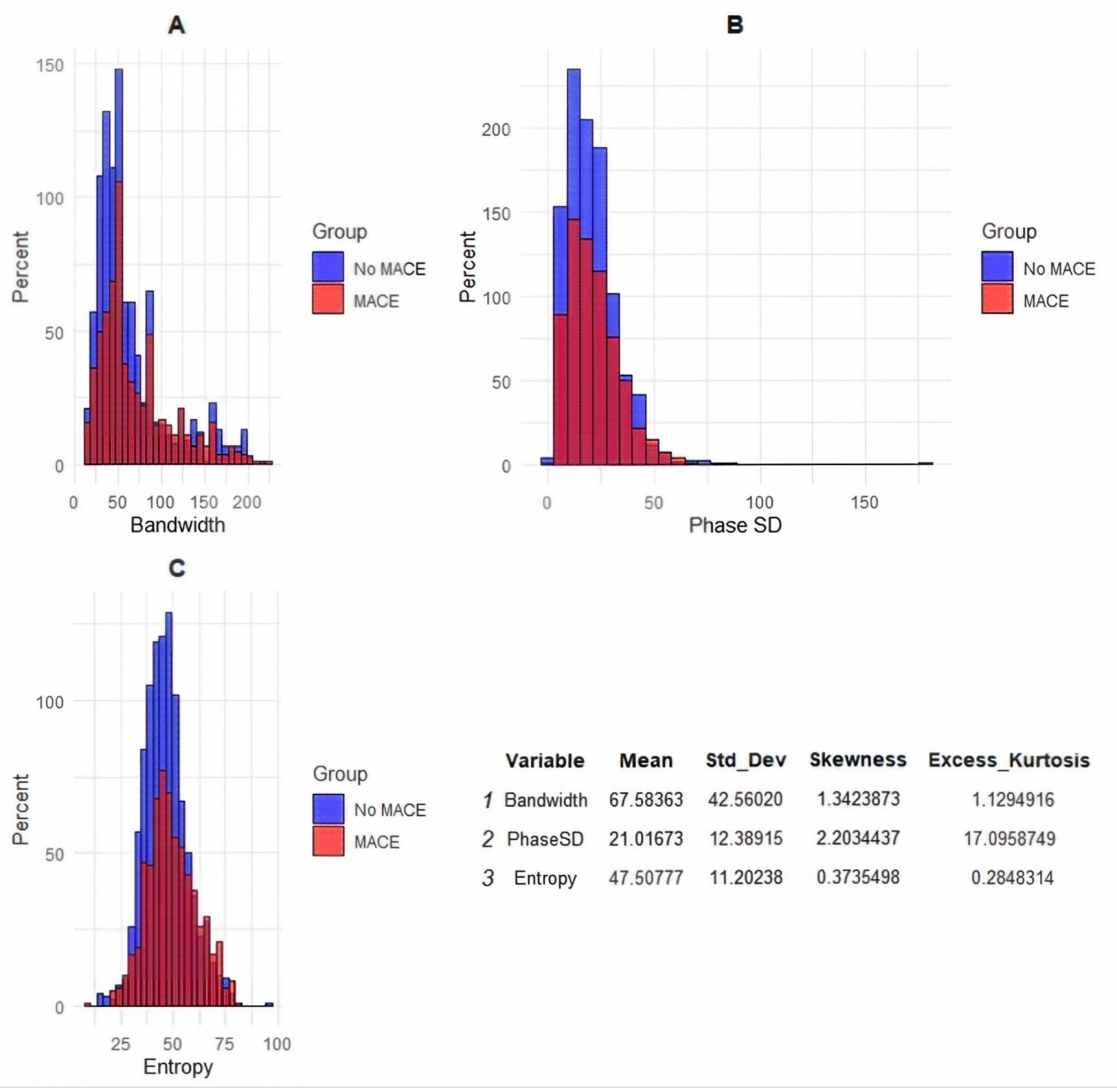
Distribution of phase variables. Histograms showing the frequency distribution of **(A)** Bandwidth, **(B)** Phase SD, and **(C)** entropy in the study cohort (*n* = 1,674). Patients are stratified by the occurrence of MACE (No MACE: *n* = 1,012; MACE: *n* = 662). Descriptive statistics, including mean, standard deviation skewness, and excess kurtosis, are provided for each variable. Normality of the distributions was assessed using the Shapiro–Wilk test. SD, standard deviation; MACE, major adverse cardiovascular events.

**Figure 3:**
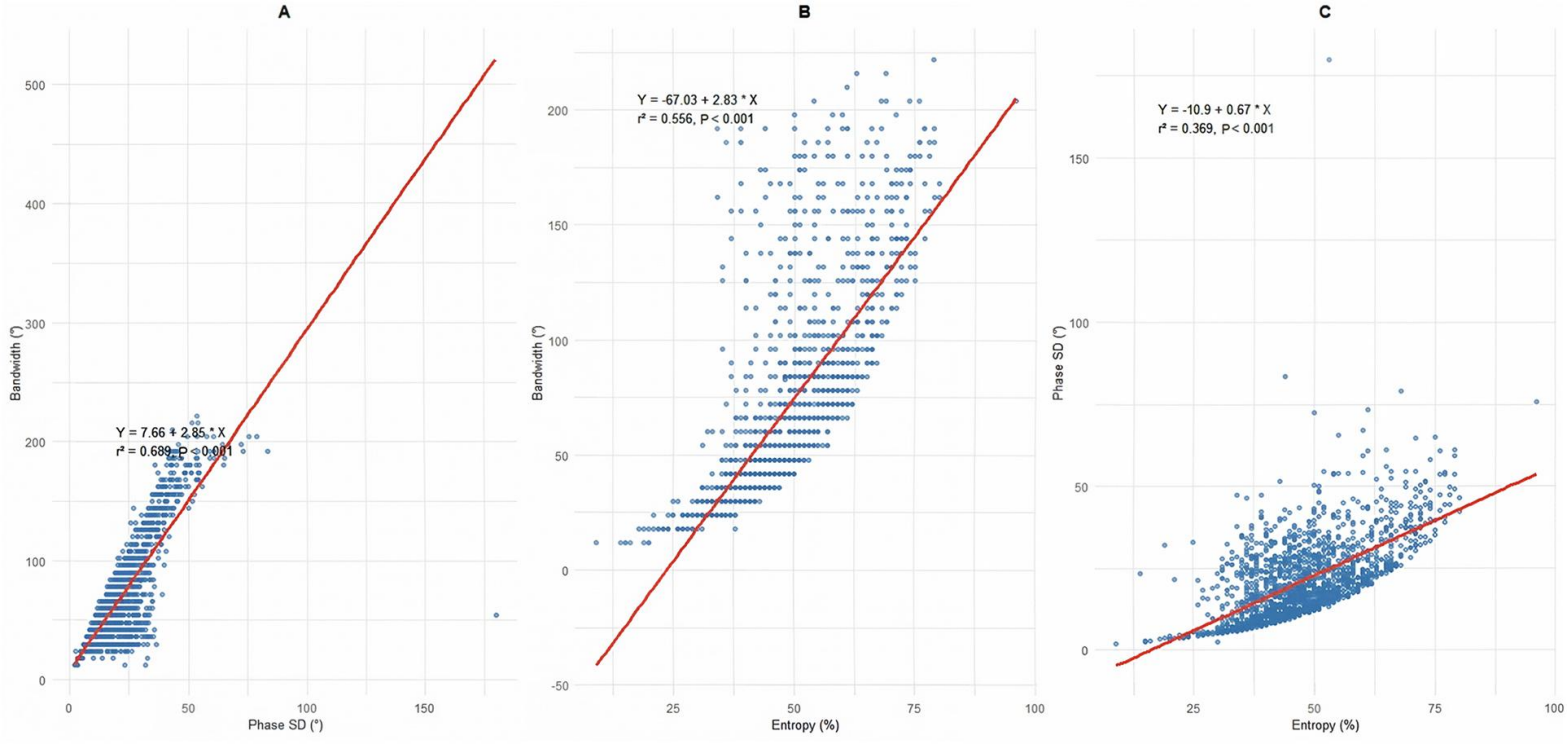
Relationship between phase analysis parameters. Scatter plots showing the relationships between **(A)** Bandwidth and Phase SD, **(B)** Bandwidth and entropy, and **(C)** Phase SD and entropy. The red lines represent the linear regression fit. (*n* = 1,674). Statistical significance was assessed using Pearson correlation analysis. All correlations were statistically significant (*P* < 0.001). SD = standard deviation.

**Figure 4:**
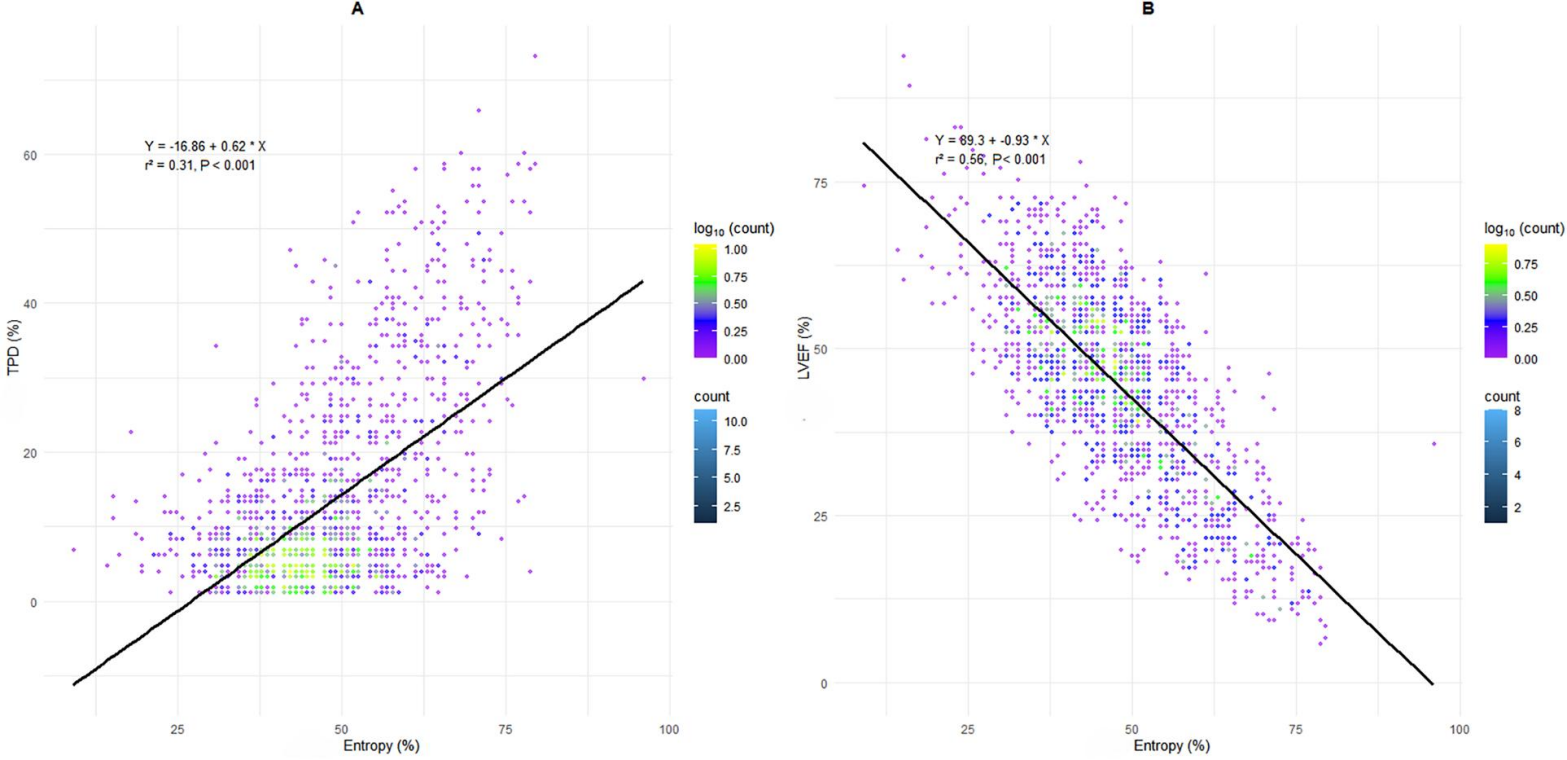
Correlation between entropy and TPD or LVEF. Scatter plots illustrating the associations between Entropy and **(A)** TPD and **(B)** LVEF. The black lines represent the linear regression fit. The color scale indicates the density of data points (log*_10_* count). (*n* = 1,674). Statistical significance was assessed using Pearson correlation analysis. Both correlations were statistically significant (*P* < 0.001). LVEF, left ventricular ejection fraction; TPD, total perfusion deficit.

The distribution of bandwidth and phase SD are highly right-skewed. In contrast, the distribution of phase entropy is quite symmetrical (Figure 2). There is a positive correlation among the three phase variables (bandwidth and phase SD *r*^2^ = 0.689, bandwidth and entropy *r*^2^ = 0.556, phase SD and entropy *r*^2^ = 0.369) (Figure 3). Entropy is positively correlated with TPD and negatively correlated with LVEF (entropy and TPD *r*^2^ = 0.31, entropy and LVEF *r*^2^ = 0.56) (Figure 4).

### Cox proportional hazards analysis

**Figure 5:**
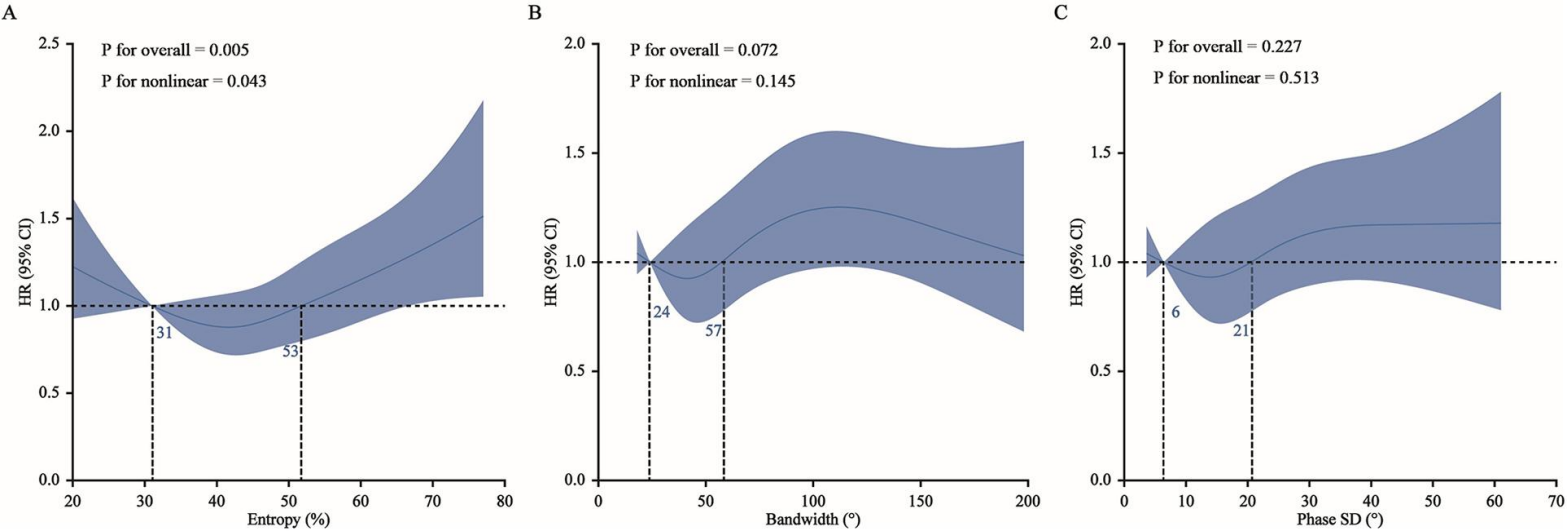
Adjusted hazard ratios for major adverse cardiovascular events of phase variables modeled with RCS. The HR (solid line) and 95%CI (shaded blue area) for major adverse cardiovascular events are plotted against increasing values of entropy **(A)**, Bandwidth **(B)**, and Phase SD **(C)** (*n* = 1,674). RCS analyses were performed to explore potential nonlinear relationships between each variable and the risk of major adverse cardiovascular events in Cox proportional hazards models. The overall and nonlinear associations were tested using likelihood ratio tests. HR and their 95% CI were estimated, with HR = 1.0 as the reference. MACE, major adverse cardiovascular events; RCS, restricted cubic splines; SD, standard deviation.

In the Cox model with restricted cubic splines, Figure 5A shows a significant nonlinear relationship between phase entropy and risk ratio, specifically manifested as a U-shaped curve (*P* < 0.05) that is, a moderate entropy value corresponds to a lower risk, while a low or high entropy value corresponds to a higher risk. The phase entropy, with its nadir hazard observed at levels between 31%–53%. To evaluate the stability and robustness of the identified phase entropy thresholds (31% and 53%), we performed internal validation using a bootstrap resampling approach with 1,000 iterations. The distribution of the derived optimal thresholds is illustrated in Supplementary Figure S7. The lower threshold demonstrated high stability, with a median value of 31% (95% CI: 31%–32%). The upper threshold showed a broader distribution, reflecting the inherent variability in high-entropy states, with a median value of 52.32% (95% CI: 42.86%–64.16%).

Therefore, in clinical applications, additional attention needs to be paid to the impact of the range of entropy values on risk. Figure 5B shows that the HR first increases and then decreases with the increase of bandwidth, presenting a nonlinear trend. the bandwidth ranged from 24°–57°. However, statistically, this relationship is not significant (*P* > 0.05). Phase SD shows a positive correlation with the hazard ratio, that is, the larger the Phase SD, the higher the hazard ratio (Figure 5C). The corresponding phase SD thresholds ranged from 6°–21°. However, statistically, this relationship is not significant (*P* > 0.05). Similar results were observed for the secondary endpoint (Supplementary Figure S3).

### The Kaplan–Meier analysis

**Figure 6:**
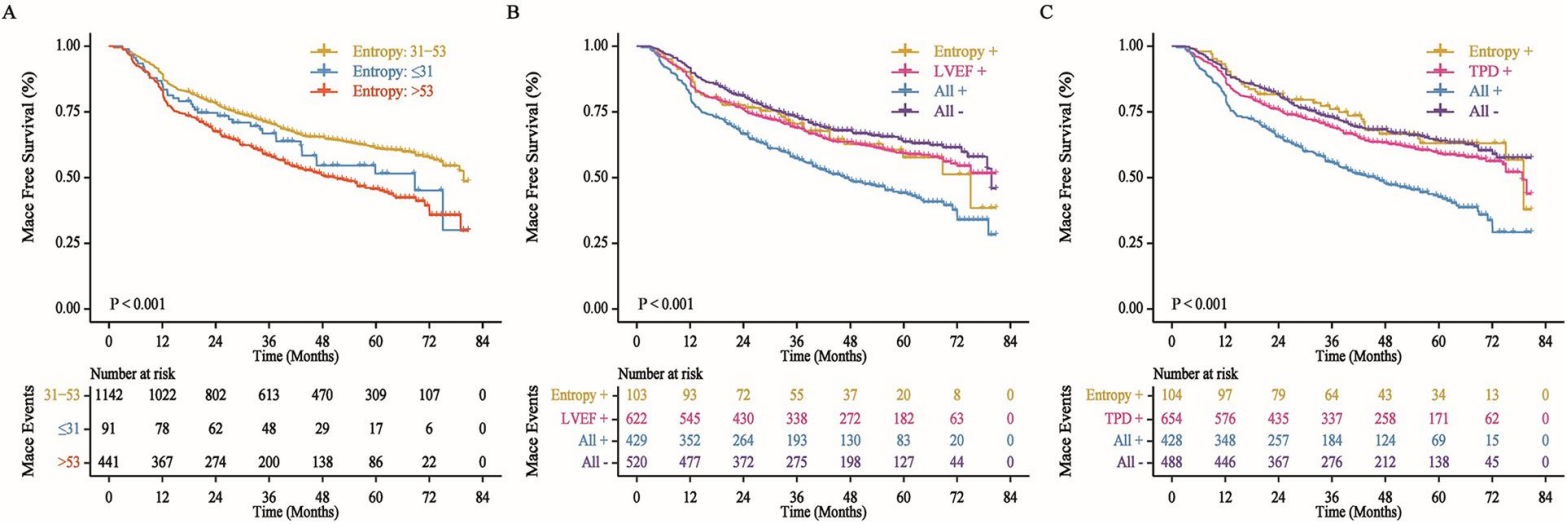
Kaplan–Meier curves for major adverse cardiovascular events by entropy, TPD and LVEF. Kaplan–Meier curves stratified by the groups based on Entropy **(A)**, LVEF and Entropy **(B)**, TPD and Entropy **(C)** are shown. (*n* = 1,674). Kaplan–Meier survival analysis was performed to estimate major adverse cardiovascular events free survival rates across groups. The log-rank test was used to compare survival distributions between strata. Patients were stratified by entropy tertiles **(A)** and combined risk categories **(B, C)** to evaluate incremental prognostic value. MACE, major adverse cardiovascular events; LVEF, left ventricular ejection fraction; TPD, total perfusion deficit.

The Kaplan–Meier curve of entropy major adverse cardiovascular events shows that people with an entropy value >53% or ≤31% may have a high risk of major adverse cardiovascular events and require closer monitoring or intervention. (Figure 6A). The Kaplan–Meier survival curves of major adverse cardiovascular events were plotted based on normal or abnormal LVEF, TPD and entropy. Entropy value >53% or ≤31% is defined as abnormal entropy value. The composite risk model demonstrated incremental prognostic stratification (*P* < 0.001 for all comparisons, log-rank test with Bonferroni correction; Figures 6B,C). Patients with all normal parameters (LVEF ≥ 50%, TPD < 5%, entropy 31%–53%) exhibited the highest major adverse cardiovascular events-free survival, while those with all abnormal parameters had the lowest survival probability. Intermediate-risk groups with isolated abnormalities in entropy, LVEF, or TPD showed intermediate event rates between these extremes. Similar results were observed for the secondary endpoint (Supplementary Figure S4).

Table 4 presents multivariable-adjusted HR with 95% CI for independent predictors of the primary and secondary endpoint. After full adjustment, phase entropy (>53% or ≤31%) was independently associated with major adverse cardiovascular events (HR 1.32, 95% CI 1.12–1.55, *P* = 0.001), whereas phase bandwidth and phase SD showed no significant association (both *P* > 0.05).

**Table 4:** Multivariable analysis of phase variables for adjusted HR of the primary and secondary endpoint.

Number	The primary endpoint Multivariable analysis		The secondary endpoint Multivariable analysis	
	Adjusted HR (95%CI)	*P*-value	Adjusted HR (95%CI)	*P*-value
Entropy (%)	1.32 (1.12–1.55)	0.001	1.34 (1.06–1.70)	0.015
Bandwidth (°)	1.14 (0.98–1.33)	0.101	1.19 (0.94–1.50)	0.139
Phase SD (°)	1.15 (0.99–1.34)	0.074	NA	NA

HR, hazard ratio; SD, standard deviation; CI, confidence interval.

### Phase analysis provides incremental prognostic value

**Figure 7:**
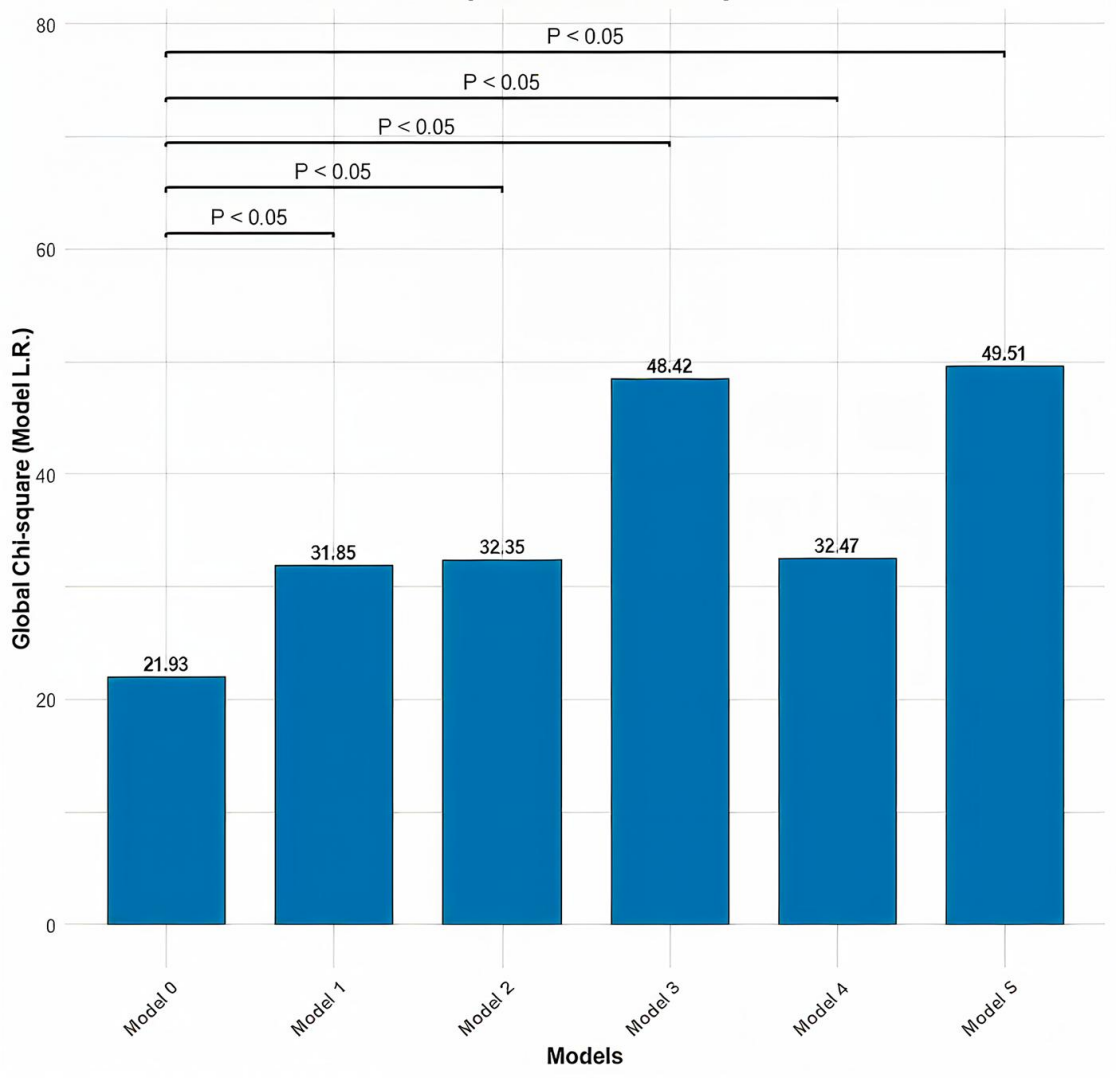
Incremental value of phase variables for prediction of major adverse cardiovascular events. Model 0: TPD; Model 1: TPD + LVEF; Model 2: TPD + LVEF + Phase SD; Model 3: TPD + LVEF + Entropy; Model 4: TPD + LVEF + Bandwidth; Model 5: TPD + LVEF + Phase SD + Entropy + Bandwidth. The incremental prognostic value of phase entropy was assessed using nested Cox proportional hazards models. The global likelihood ratio chi-square test was used to compare model fit across specifications, with *P* < 0.05 considered statistically significant. (*n* = 1,674). MACE, major adverse cardiovascular events; SD, standard deviation; LVEF, left ventricular ejection fraction; TPD, total perfusion deficit.

Figure 7 shows the global results of major adverse cardiovascular events prediction. The overall *χ*^2^ of Model1 (TPD + LVEF) was significantly increased compared with Model 0 (TPD)（*P* < 0.05). Compared with Model 1, the global *χ*^2^ of the models with phase variables (Model 2: TPD + LVEF + Phase SD; Model 3: TPD + LVEF + Entropy; Model 4: TPD + LVEF + Bandwidth) significantly increased, indicating that when phase variables were added to TPD and LVEF, Phase variables provide incremental diagnostic value (*P* < 0.05) (Figure 7). Model 5 (TPD + LVEF + Phase SD + Entropy + Bandwidth) had the highest *χ*^2^ value (*P* < 0.05). Similar results were observed for the secondary endpoint (Supplementary Figure S5).

**Figure 8:**
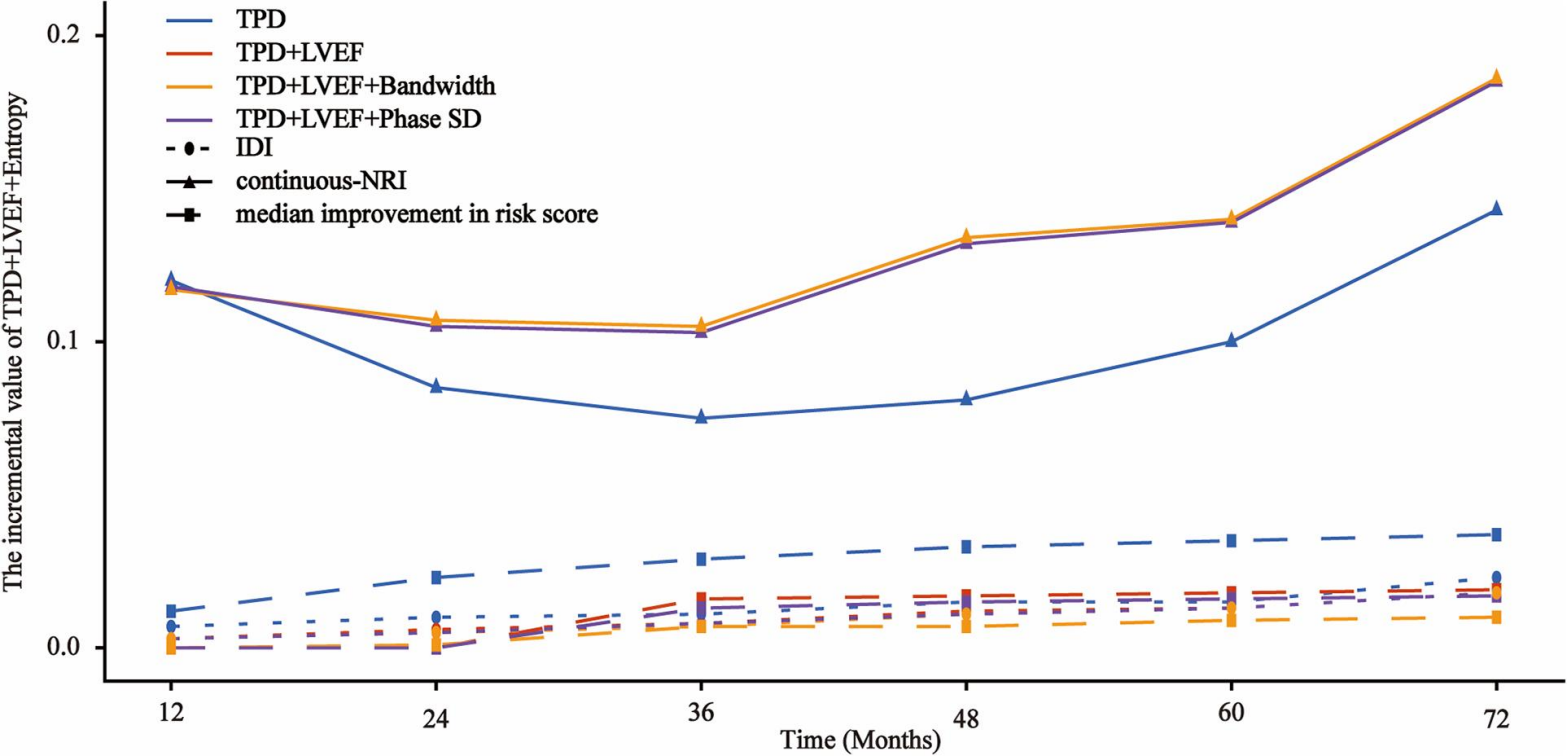
NRI and IDI statistics for the addition of SPECT variables to major adverse cardiovascular events prediction. Time-dependent ROC curve analysis was performed to assess the incremental prognostic value of each variable. The C-index (concordance index), IDI, continuous NRI and median risk score improvement were calculated to quantify the improvement in predictive performance of nested models over baseline models. (*n* = 1,674). IDI, integrated discrimination improvement; NRI, net reclassification improvement; SD, standard deviation; LVEF, left ventricular ejection fraction; TPD, total perfusion deficit.

Using continuous NRI and IDI indices, we evaluated incremental prognostic value of the model with TPD and LVEF (Model 1) significantly improved risk classification for major adverse cardiovascular events compared to the model with TPD (Model 0). The risk model with entropy Model 3 improved prediction of major adverse cardiovascular events compared to the model with TPD and LVEF (Model 1) but not for the models with phase SD and bandwidth (Model 2, Model 4) (Figure 8, Supplementary Table S1). Phase entropy uniquely contributed to refined major adverse cardiovascular events risk stratification beyond conventional parameters. Similar results were observed for the secondary endpoint (Supplementary Figure S6).

The time-dependent AUC curves of the Cox regression models are shown in Supplementary Figure S8. The model 0 showed the lowest discriminative ability across all follow-up time points (6–72 months). Compared with the model 0, the addition of conventional parameters (Model 1) resulted in a modest improvement in AUC. Models incorporating phase-related parameters, particularly phase entropy (Model 3) and the full model with all phase features (Model 5), consistently achieved higher AUC values at all time points. The full model including phase entropy showed the best long-term predictive performance, with the 72-month AUC reaching approximately 0.62, compared with 0.57 for the model 0.To further validate the clinical utility of phase entropy, we assessed the calibration performance of the prognostic models using calibration curves at 12, 36, and 60 months (Supplementary Figure S9). The model incorporating phase entropy (Model 3) demonstrated superior calibration compared to the baseline model (Model 1), with predicted probabilities closely aligning with observed major adverse cardiovascular events rates across the entire risk spectrum.

The DCA results are shown in Supplementary Figure S10, evaluating the net benefit of each model across clinically relevant risk thresholds (10–60%). Compared with the model 0 and model 1, models including phase entropy (Model 3 and Model 5) consistently yielded higher net benefit. The greatest improvement in net benefit was observed at intermediate risk thresholds (20–40%), where phase entropy-guided risk stratification provided the largest clinical advantage.

We performed risk reclassification analysis stratified by 72-month cumulative major adverse cardiovascular events probability, with three predefined risk strata: low risk (< 20%), intermediate risk (20%–40%), and high risk (≥ 40%). Reclassification shifts between model 1 (TPD + LVEF) and model 3 (TPD + LVEF + Entropy) are summarized in Supplementary Table S2.

No patients were classified into the low-risk group across all subgroups. In the overall cohort, 592 patients were initially categorized as intermediate risk by the model 1; of these individuals, 266 remained intermediate risk and 326 were reclassified to high risk after adding phase entropy. All 1,082 patients with baseline high risk retained the same risk category in the model 3.

Stratified by clinical outcomes, among 191 intermediate-risk patients who experienced major adverse cardiovascular events, 78 stayed in the intermediate stratum and 113 were upgraded to high risk. Among 58 intermediate-risk patients without major adverse cardiovascular events, 22 remained intermediate risk and 36 were reclassified as high risk. All baseline high-risk patients showed consistent risk assignments in both models.

## Discussion

Our study revealed a U-shaped relationship between phase entropy and prognosis, as demonstrated by the hazard ratio curve (Figure 5). Phase entropy, bandwidth, TPD and LVEF are potential biomarkers for predicting major adverse cardiovascular events (Table 2). However, when analyzed continuously, only phase entropy retained a significant association with major adverse cardiovascular events (Table 4). Furthermore, incorporating phase entropy information significantly improved the discriminatory power for major adverse cardiovascular events prediction beyond that provided by TPD and LVEF alone (Figure 7).

Ventricular systolic synchrony plays a critical role in maintaining optimal cardiac function. Previous studies have demonstrated a positive correlation between elevated entropy values and an increased risk of major adverse cardiovascular events (4). This association may be explained by two potential mechanisms. First, impaired coordination of ventricular motion may reduce global myocardial contractility. Second, ventricular dyssynchrony can prolong the myocardial effective refractory period and shorten diastole, thereby reducing ventricular filling time and coronary perfusion duration, which ultimately compromises myocardial perfusion (23).

Limited literature has reported an association between reduced entropy and major adverse cardiovascular events. Studies have demonstrated that extensive microvascular injuries, such as those observed in diabetes and post-COVID-19 sequelae, result in decreased overall perfusion homogeneity (manifested as low entropy), which, despite being asymptomatic, significantly elevates the risk of major adverse cardiovascular events when the heart-to-mediastinum ratio is ≤1.6 (24). In aging hearts, diffuse fibrosis gradually replaces normal myocardial tissue, thereby diminishing perfusion uniformity and lowering entropy values. This condition is often accompanied by impaired cardiac reserve function and increased susceptibility to irreversible damage during myocardial ischemia-reperfusion injury (25). Abnormal cardiac sympathetic nerve activity, such as dysregulation of the BNST-ARC-NTS neural circuit, can disrupt global perfusion regulation, which is reflected as reduced entropy. This phenomenon concurrently activates pathways related to anxiety and hyperglycemia, thereby indirectly increasing the risk of cardiovascular diseases (26).

Phase analysis typically incorporates parameters such as bandwidth, phase SD, and entropy. Among these, entropy serves as a measure of the complexity and disorder in phase distribution. In contrast, conventional parameters like phase bandwidth and standard deviation only reflect the extent of dispersion within a given time frame (27, 28). However, most prior prognostic studies have predominantly utilized bandwidth and phase SD as primary variables (29, 30), suggesting that the clinical utility of entropy warrants further investigation.

Assessment of ventricular systolic synchrony is critical for guiding treatment selection and predicting therapeutic outcomes in CRT. While radionuclide MPI phase analysis is an established methodology, existing evidence primarily focuses on phase SD and bandwidth (31, 32). Incorporating phase entropy provides complementary information that enables a more comprehensive evaluation of LVMD. This approach enhances identification of optimal CRT candidates, facilitates treatment optimization, and improves prediction accuracy of CRT response, ultimately enhancing patient clinical outcomes.

We established optimal diagnostic thresholds (Entropy: 31%–53%; Bandwidth: 24°–57°; Phase SD: 6°–21°),while these thresholds were derived from our cohort, they require further validation in independent, prospective, multi-center datasets before clinical implementation.Phase entropy is derived from Shannon information entropy applied to cardiac phase distributions, conferring intrinsic resistance to local outliers through probability-weighted integration (33). Clinically, this contrasts sharply with phase SD based metrics, whose susceptibility to extreme values renders them unreliable in noisy environments such as transthoracic echocardiography with respiratory motion artifacts (19, 27). Phase entropy assesses the coordination of myocardial contraction more comprehensively by analyzing the information diversity of the entire distribution.

Bandwidth and phase SD exhibited markedly right-skewed distributions (skewness > 1), whereas entropy followed a normal distribution (Figure 2). Notably, phase entropy was the only continuous variable independently predictive of major adverse cardiovascular events (adjusted HR 1.32, 95% CI 1.12–1.55, *P* = 0.001; Table 4). These results establish entropy as a superior predictor of major adverse cardiovascular events compared to bandwidth or phase SD. In SPECT MPI phase analysis, entropy quantifies spatiotemporal patterns of LVMD, offering clinical value through three key aspects: Diagnostic utility in detecting subclinical contraction abnormalities; Enhanced prognostic capability beyond traditional perfusion/function parameters; Support for clinical decision-making in selecting candidates for CRT.

Previous studies have established left ventricular entropy derived from cardiac magnetic resonance as a robust independent predictor of major adverse cardiovascular events in patients with CAD. In a cohort of 314 CAD patients, 110 developed major adverse cardiovascular events over a median follow-up of 13 months. The risk of major adverse cardiovascular events was significantly elevated in the high entropy group (*P* < 0.001) (34). Similar findings have been reported in studies involving patients with various other cardiac conditions (2, 35, 36).

In the largest imaging study to date, phase variables were independently associated with major adverse cardiovascular events and enhanced risk stratification beyond that achievable by perfusion and LVEF alone. Notably, only entropy remained independently predictive of major adverse cardiovascular events. The inclusion of phase entropy significantly improved the model's discriminatory power for predicting major adverse cardiovascular events when added to TPD and LVEF (*P* < 0.001) (4).

A meta-analysis revealed that among SPECT-derived LVMD parameters, high phase SD, bandwidth, and phase entropy were consistently associated with increased risks of all-cause mortality, cardiac death, and major adverse cardiovascular events. Studies that combined multiple phase parameters to define LVMD yielded HR compared to those using phase entropy alone (*P* = 0.018) (37).

In a study of patients with severe heart failure (New York Heart Association class III–IV, LVEF ≤ 35%, and prolonged QRS duration ≥ 120 ms), bandwidth and phase SD threshold of 72.5° and 19.6°, respectively, demonstrated high accuracy in predicting response to CRT (38). The progressive decrease in phase entropy often parallels the worsening severity of heart failure (as classified by NYHA criteria), indicating that the disease has entered an advanced stage and the heart's functional reserve is depleted (39). Whether phase entropy is elevated or reduced, it reflects a pathological state of the cardiac autonomic nervous system (40) and is associated with an increased cardiovascular risk. In this study, we found that the mortality rate of patients with a decrease in entropy value ≤ 31% was also increased. Phase entropy exhibited a U-shaped association with major adverse cardiovascular events, indicating that both extremely low and high entropy values reflect pathological conditions. High entropy was closely correlated with evident mechanical dyssynchrony and systolic dysfunction, whereas the mechanisms underlying the elevated risk in patients with low entropy (≤31) remain poorly understood. Patients in the low-entropy subgroup had preserved left ventricular ejection fraction and mild myocardial perfusion impairment, implying their increased major adverse cardiovascular events risk was not attributable to advanced cardiac dysfunction or severe ischemia. Further comparison between the low and normal entropy groups confirmed this unique clinical phenotype. Notably, low entropy may represent a distinct subclinical pathological state. We hypothesize that low entropy may indicate early microvascular lesions or subtle myocardial changes undetectable by conventional imaging. Since the present study lacks direct histopathological evidence to validate the underlying mechanisms, these interpretations are proposed as hypotheses for future research.The phase entropy combined with traditional parameters (LVEF, TPD) can optimize the risk prediction model and guide individualized intervention. The spatiotemporal resolution of entropy value calculation is improved through dynamic SPECT/CT fusion imaging. Through artificial intelligence texture analysis, the entropy value change pattern can be automatically identified and subclinical lesions can be warned (24). We assessed dyssynchrony using rest scan and stress phase variables were not analyzed. Abnormalities in LVEF, TPD and entropy have a significant impact on the survival rate of patients, especially when all three are abnormal, the survival rate of patients is significantly reduced. Clinically, this analysis result can be utilized to carry out targeted management and intervention for patients in different risk groups (Figure 6).

This study highlights the added value of phase variables in predicting major adverse cardiovascular events beyond that provided by conventional parameters such as TPD and LVEF, thereby improving risk stratification. The incremental prognostic value of phase entropy was comprehensively validated using global *χ*^2^, NRI, and IDI, time-dependent AUC, calibration curves, and decision curve analysis. The global chi-square analysis showed that adding phase entropy resulted in the largest improvement in model fit compared with other phase parameters, indicating that it contributed the most to explaining the variance in MACE risk. The continuous-NRI and IDI results confirmed that phase entropy provided significant incremental predictive value over time, particularly for long-term risk prediction.

Time-dependent AUC analysis demonstrated that models incorporating phase entropy outperformed conventional models in predicting major adverse cardiovascular events risk across the entire follow-up period, particularly improving long-term predictive performance. This indicates that phase entropy provides additional prognostic information beyond traditional SPECT parameters, which may be attributed to its ability to capture subtle mechanical dyssynchrony not reflected in conventional perfusion or functional metrics. The 0.05 increase in 72-month time-dependent AUC (from 0.57 to 0.62) is considered clinically meaningful within the context of long-term CAD prognostic research using gated SPECT-MPI. Long-duration follow-up inevitably attenuates model discriminative power, and most established SPECT-based prognostic models report AUC values between 0.55 and 0.65 at 6-year follow-up. According to accepted criteria in cardiovascular research, an AUC rise of ≥0.03 denotes a worthwhile clinical improvement for chronic disease risk models, and our 0.05 increment meets this standard.

Calibration curves confirmed that the addition of phase entropy did not compromise model calibration, with predicted probabilities closely matching observed event rates. This finding is critical for clinical application, as it ensures that risk estimates derived from the model are reliable and interpretable.

Furthermore, DCA revealed that models including phase entropy provided higher net benefit across a wide range of clinically relevant risk thresholds, especially at intermediate probabilities where clinical decision-making is most challenging. This highlights the potential of phase entropy to improve risk stratification and guide clinical management decisions, such as intensified follow-up or preventive interventions.

Collectively, these results support that phase entropy enhances both the statistical performance and clinical utility of prognostic models, reinforcing its role as a valuable imaging biomarker in risk stratification for patients. Gated SPECT-MPI delivers significant prognostic value in CAD management. Its phase-derived parameters are automatically quantified, demonstrate high reproducibility, and impose no additional costs. Automatically include phase entropy in SPECT-MPI reports.

## Limitations

Several limitations of this study should be acknowledged. A limitation of this study is the exclusive reliance on resting gated SPECT-MPI, without the assessment of stress-induced phase variables. While stress-induced mechanical dyssynchrony can unmask ischemia-related abnormalities that are not apparent at rest, our study cohort included a significant proportion of patients who were ineligible for exercise or pharmacological stress testing due to physical limitations or comorbidities. In such clinical settings, resting gated SPECT-MPI often serves as the primary imaging modality. Our findings demonstrate that resting phase entropy provides robust, independent prognostic information in this population, capturing baseline mechanical heterogeneity such as chronic myocardial remodeling or diffuse fibrosis that persists regardless of the acute ischemic state. Nevertheless, future studies incorporating stress phase analysis are warranted to further elucidate the incremental value of stress-induced dyssynchrony in patients capable of undergoing stress testing. The single-center design may introduce selection bias. Notably, when excluding patients with diabetes and PVD to assess the robustness of the low-entropy phenotype, the sample size of the low-entropy subgroup was reduced to seven cases. While a formal sensitivity analysis was underpowered, a descriptive assessment confirmed that these patients consistently exhibited the same phenotype preserved LVEF, minimal perfusion defects, and near-normal mechanical synchrony suggesting that the low-entropy state is not an artifact of metabolic or vascular comorbidities, but rather a distinct clinical entity. However, the underlying mechanism in the absence of these comorbidities warrants further validation in larger, prospective cohorts. Second, our study lacks direct histopathological or cardiac magnetic resonance (CMR) mapping evidence to definitively characterize the myocardial remodeling associated with low entropy. While our findings suggest a unique myocardial phenotype, future multi-modal imaging studies, specifically those integrating gated SPECT-MPI with CMR tissue characterization, are essential to elucidate the precise structural and functional substrates of this low-entropy state.

Furthermore, we did not collect QRS duration data, which is a standard marker for electrical conduction disease. While electrical dyssynchrony is a primary driver of mechanical dyssynchrony, it is not the sole determinant. Phase entropy, as derived from gated SPECT-MPI, quantifies the spatial heterogeneity of myocardial contraction, which can arise from various non-electrical substrates such as diffuse myocardial fibrosis, regional wall motion abnormalities, and microvascular dysfunction. Consequently, phase entropy captures a distinct dimension of myocardial vulnerability that is not fully represented by QRS duration alone. Although we cannot definitively exclude the potential influence of underlying conduction disease on our findings, our results suggest that phase entropy provides prognostic information that is complementary to, rather than redundant with, electrical conduction parameters. Future prospective studies are warranted to simultaneously collect QRS duration and phase entropy to further elucidate their independent and synergistic roles in electromechanical coupling and risk stratification. Previous research suggests that the association between mechanical dyssynchrony measured by gated SPECT and major adverse cardiovascular events is stronger than the association between QRS duration and major adverse cardiovascular events (30). Future studies may concurrently assess QRS duration and phase entropy to elucidate their independent and synergistic prognostic implications. All analyses were performed using Cedars-Sinai's QGS and QPS software. However, the generalizability of this approach to software from other vendors requires further investigation.

It is noteworthy that our study cohort includes a significant proportion of patients with preserved LVEF (≥50%), a population where HF with preserved ejection fraction (HFpEF) is highly prevalent among those with underlying coronary artery disease. Relying solely on LVEF to identify high-risk individuals in this subgroup is inherently limited, as these patients often exhibit significant cardiovascular risk despite normal systolic function. Our findings suggest that phase entropy serves as a valuable auxiliary imaging biomarker in this context. By capturing subtle mechanical dyssynchrony that persists even when LVEF is preserved, phase entropy provides incremental prognostic information that complements conventional functional metrics. Future studies are warranted to specifically evaluate the diagnostic and prognostic utility of phase entropy in the distinct clinical phenotype of HFpEF.

## Conclusion

A significant nonlinear association was identified between phase entropy and major adverse cardiovascular events. Distinct values of entropy were associated with varying survival outcomes. In comparison to bandwidth, phase entropy exhibited a superior predictive capability for major adverse cardiovascular events. These phase parameters can be quantified automatically, thereby providing an objective foundation for clinical risk stratification. Prospective studies are necessary to elucidate the underlying pathological mechanisms and to confirm the comprehensive clinical significance of phase entropy.

## Data Availability

The raw data supporting the conclusions of this article will be made available by the authors, without undue reservation.

## Supplementary material

Supplementary Figure S1 Annualized incidence of major adverse cardiovascular events for Bandwidth. The bar chart illustrates the annualized major adverse cardiovascular events incidence rates (%) across ten deciles of bandwidth (*n* = 1,674 total patients; *n* = 99–288 per decile). The orange line represents the mean entropy value for each decile. Annualized major adverse cardiovascular events rates were calculated as events per 100 person-years. Statistical comparisons across deciles were performed using the *χ*^2^ test for categorical variables. MACE, major adverse cardiovascular events; LVEF, left ventricular ejection fraction; TPD, total perfusion deficit.

Supplementary Figure S2 Annualized incidence of major adverse cardiovascular events for Phase SD. The bar chart illustrates the annualized major adverse cardiovascular events incidence rates (%) across ten deciles of phase SD (*n* = 1,674 total patients; *n* = 163–173 per decile). The orange line represents the mean entropy value for each decile. Annualized major adverse cardiovascular events rates were calculated as events per 100 person-years. Statistical comparisons across deciles were performed using the *χ*^2^ test for categorical variables. MACE, major adverse cardiovascular events; LVEF, left ventricular ejection fraction; SD, standard deviation; TPD, total perfusion deficit.

Supplementary Figure S3 Adjusted hazard ratios for hard events of phase variables modeled with RCS. HR are shown on the *Y*-axis and the comparison of each phase variable, with the dashed line indicating the 95%CI, to the mean reference value in the first quartile is shown on the *X*-axis. (*n* = 1,674). RCS analyses were performed to explore potential nonlinear relationships between each variable and the risk of major adverse cardiovascular events in multivariable-adjusted Cox proportional hazards models. The overall and nonlinear associations were tested using likelihood ratio tests. HR and their 95% CI were estimated, with HR = 1.0 as the reference. MACE, major adverse cardiovascular events; RCS, restricted cubic splines; SD, standard deviation.

Supplementary Figure S4 Kaplan–Meier curves for hard events by Entropy, TPD and LVEF. Kaplan–Meier curves stratified by the groups based on entropy (A), LVEF and entropy (B), TPD and Entropy (C) are shown. (*n* = 1,674). Kaplan–Meier survival analysis was performed to estimate major adverse cardiovascular events free survival rates across groups. The log-rank test was used to compare survival distributions between strata. Patients were stratified by phase entropy tertiles (A) and combined risk categories (B and C) to evaluate incremental prognostic value after adjustment for covariates. MACE, major adverse cardiovascular events; LVEF, left ventricular ejection fraction; TPD, total perfusion deficit.

Supplementary Figure S5 Incremental value of phase variables for prediction of hard events. Model 0: TPD; Model 1: TPD + LVEF; Model 2: TPD + LVEF + Phase SD; Model 3: TPD + LVEF + Entropy; Model 4: TPD + LVEF + Bandwidth; Model 5: TPD + LVEF + Phase SD + Entropy + Bandwidth. The incremental prognostic value of phase entropy was assessed using nested multivariable-adjusted Cox proportional hazards models. The global likelihood ratio chi-square test was used to compare model fit across specifications, with *P* < 0.05 considered statistically significant. (*n* = 1,674). MACE, major adverse cardiovascular events; SD, standard deviation; LVEF, left ventricular ejection fraction; TPD, total perfusion deficit.

Supplementary Figure S6 NRI and IDI statistics for the addition of SPECT variables to hard events prediction. Time-dependent ROC curve analysis was performed to assess the incremental prognostic value of each variable in multivariable-adjusted models. The C-index, IDI, continuous NRI and median risk score improvement were calculated to quantify the improvement in predictive performance of nested models over baseline models. (*n* = 1,674). IDI, integrated discrimination improvement; NRI, net reclassification improvement; SD, standard deviation; LVEF, left ventricular ejection fraction; TPD, total perfusion deficit.

Supplementary Figure S7 Distribution of Optimal Entropy thresholds derived from 1,000 Bootstrap replications. The histograms display the frequency distribution of the lower (left) and upper (right) prognostic thresholds for phase entropy. Red dashed lines indicate the median values, and dotted lines represent the 95%CI. The lower threshold shows high consistency (median 31%), while the upper threshold (median 52.32%) demonstrates the distribution of optimal cut-offs across resampled datasets.

Supplementary Figure S8 Time-dependent AUC of Cox regression models for major adverse cardiovascular events prediction. The figure illustrates the predictive performance (AUC) of different models over a 72-month follow-up period (*n* = 1,674). Models were constructed as follows: Model 0 (TPD), Model 1 (TPD + LVEF), Model 2 (TPD + LVEF + Phase SD), Model 3 (TPD + LVEF + Entropy), Model 4 (TPD + LVEF + Bandwidth), and Model 5 (TPD + LVEF + Phase SD + Entropy + Bandwidth). The model incorporating all features (Model 5) consistently demonstrated the highest predictive accuracy across all time points. Statistical comparisons of AUC values were performed using the DeLong test.

Supplementary Figure S9 Calibration curves for major adverse cardiovascular events prediction at 12, 36, and 60 months. The calibration plots compare the predicted major adverse cardiovascular events probability (*x*-axis) with the observed major adverse cardiovascular events frequency (*y*-axis). The diagonal dashed line represents the ideal calibration. The model incorporating phase entropy (Model 3, gold line) shows excellent agreement between predicted and observed risks, demonstrating superior performance compared to the baseline model (Model 1, red line). (*n* = 1,674; 662 events; B = 200 bootstrap resamples).

Supplementary Figure S10 DCA for major adverse cardiovascular events prediction. The DCA plots compare the net benefit of different models (model 0, model 1, model 2 and model 4) across a range of risk thresholds (*n* = 1,674). The *y*-axis represents the net benefit, and the *x*-axis represents the threshold probability. The model incorporating phase entropy (model 3) demonstrates the highest net benefit across the clinically relevant threshold range. Statistical significance was assessed using the likelihood ratio test.
