# Supplementary material for: Phase entropy of gated SPECT-MPI for predicting major adverse cardiovascular events: incremental prognostic value beyond perfusion and function
Source: Front Cardiovasc Med. 2026 Jul 7;13:1839622. doi: 10.3389/fcvm.2026.1839622 (PMC13385191; doi:10.3389/fcvm.2026.1839622)
Supplement: Supplementary file 1 [file Datasheet1.docx]

**Supplementary Table 1: Time-dependent AUC values for prognostic models at different follow-up time points.**

| **Variables** | **Time (months)** | **Model** | **Estimate** | **95% CI Lower** | **95% CI Upper** | ***P*-value** |
| --- | --- | --- | --- | --- | --- | --- |
| TPD | 12 | M1 | 0.007 | 0.002 | 0.014 | 0.004 |
| TPD | 12 | M2 | 0.120 | 0.054 | 0.194 | 0.000 |
| TPD | 12 | M3 | 0.012 | 0.000 | 0.019 | 0.000 |
| TPD+LVEF | 12 | M1 | 0.003 | 0.000 | 0.009 | 0.012 |
| TPD+LVEF | 12 | M2 | 0.117 | 0.047 | 0.184 | 0.004 |
| TPD+LVEF | 12 | M3 | 0.000 | 0.000 | 0.010 | 0.663 |
| 0.663TPD+LVEF+Phase SD | 12 | M1 | 0.003 | 0.000 | 0.009 | 0.028 |
| TPD+LVEF+Phase SD | 12 | M2 | 0.118 | -0.022 | 0.184 | 0.084 |
| TPD+LVEF+Phase SD | 12 | M3 | 0.000 | 0.000 | 0.009 | 0.579 |
| TPD+LVEF+Phase Bandwidth | 12 | M1 | 0.003 | 0.000 | 0.007 | 0.052 |
| TPD+LVEF+Phase Bandwidth | 12 | M2 | 0.117 | 0.019 | 0.178 | 0.012 |
| TPD+LVEF+Phase Bandwidth | 12 | M3 | 0.000 | -0.002 | 0.007 | 0.850 |
| TPD | 24 | M1 | 0.010 | 0.004 | 0.019 | 0.000 |
| TPD | 24 | M2 | 0.085 | 0.034 | 0.155 | 0.000 |
| TPD | 24 | M3 | 0.023 | 0.000 | 0.033 | 0.000 |
| TPD+LVEF | 24 | M1 | 0.006 | 0.001 | 0.014 | 0.008 |
| TPD+LVEF | 24 | M2 | 0.107 | 0.052 | 0.159 | 0.004 |
| TPD+LVEF | 24 | M3 | 0.000 | 0.000 | 0.018 | 0.882 |
| TPD+LVEF+Phase SD | 24 | M1 | 0.005 | 0.000 | 0.013 | 0.052 |
| TPD+LVEF+Phase SD | 24 | M2 | 0.105 | -0.010 | 0.157 | 0.068 |
| TPD+LVEF+Phase SD | 24 | M3 | 0.000 | 0.000 | 0.017 | 0.683 |
| TPD+LVEF+Phase Bandwidth | 24 | M1 | 0.005 | 0.000 | 0.012 | 0.024 |
| TPD+LVEF+Phase Bandwidth | 24 | M2 | 0.107 | 0.026 | 0.156 | 0.012 |
| TPD+LVEF+Phase Bandwidth | 24 | M3 | 0.001 | -0.003 | 0.013 | 0.315 |
| TPD | 36 | M1 | 0.011 | 0.004 | 0.023 | 0.000 |
| TPD | 36 | M2 | 0.075 | 0.029 | 0.148 | 0.000 |
| TPD | 36 | M3 | 0.029 | 0.000 | 0.041 | 0.000 |
| TPD+LVEF | 36 | M1 | 0.008 | 0.001 | 0.017 | 0.004 |
| TPD+LVEF | 36 | M2 | 0.105 | 0.054 | 0.153 | 0.004 |
| TPD+LVEF | 36 | M3 | 0.016 | 0.000 | 0.023 | 0.012 |
| TPD+LVEF+Phase SD | 36 | M1 | 0.008 | 0.001 | 0.017 | 0.008 |
| TPD+LVEF+Phase SD | 36 | M2 | 0.103 | 0.028 | 0.149 | 0.012 |
| TPD+LVEF+Phase SD | 36 | M3 | 0.013 | 0.000 | 0.021 | 0.012 |
| TPD+LVEF+Phase Bandwidth | 36 | M1 | 0.007 | 0.001 | 0.016 | 0.012 |
| TPD+LVEF+Phase Bandwidth | 36 | M2 | 0.105 | 0.024 | 0.150 | 0.012 |
| TPD+LVEF+Phase Bandwidth | 36 | M3 | 0.007 | 0.000 | 0.017 | 0.044 |
| TPD | 48 | M1 | 0.015 | 0.005 | 0.030 | 0.000 |
| TPD | 48 | M2 | 0.081 | 0.028 | 0.181 | 0.008 |
| TPD | 48 | M3 | 0.033 | 0.000 | 0.046 | 0.000 |
| TPD+LVEF | 48 | M1 | 0.012 | 0.003 | 0.025 | 0.000 |
| TPD+LVEF | 48 | M2 | 0.134 | 0.076 | 0.186 | 0.000 |
| TPD+LVEF | 48 | M3 | 0.017 | 0.000 | 0.026 | 0.000 |
| TPD+LVEF+Phase SD | 48 | M1 | 0.011 | 0.002 | 0.024 | 0.008 |
| TPD+LVEF+Phase SD | 48 | M2 | 0.132 | 0.053 | 0.177 | 0.004 |
| TPD+LVEF+Phase SD | 48 | M3 | 0.015 | 0.000 | 0.024 | 0.000 |
| TPD+LVEF+Phase Bandwidth | 48 | M1 | 0.011 | 0.003 | 0.023 | 0.000 |
| TPD+LVEF+Phase Bandwidth | 48 | M2 | 0.134 | 0.051 | 0.185 | 0.008 |
| TPD+LVEF+Phase Bandwidth | 48 | M3 | 0.007 | 0.000 | 0.020 | 0.020 |
| TPD | 60 | M1 | 0.015 | 0.005 | 0.033 | 0.008 |
| TPD | 60 | M2 | 0.100 | 0.031 | 0.193 | 0.004 |
| TPD | 60 | M3 | 0.035 | 0.000 | 0.049 | 0.000 |
| TPD+LVEF | 60 | M1 | 0.013 | 0.003 | 0.030 | 0.000 |
| TPD+LVEF | 60 | M2 | 0.140 | 0.075 | 0.201 | 0.000 |
| TPD+LVEF | 60 | M3 | 0.018 | 0.000 | 0.028 | 0.004 |
| TPD+LVEF+Phase SD | 60 | M1 | 0.013 | 0.002 | 0.028 | 0.012 |
| TPD+LVEF+Phase SD | 60 | M2 | 0.139 | 0.042 | 0.196 | 0.004 |
| TPD+LVEF+Phase SD | 60 | M3 | 0.016 | 0.000 | 0.026 | 0.000 |
| TPD+LVEF+Phase Bandwidth | 60 | M1 | 0.013 | 0.003 | 0.027 | 0.008 |
| TPD+LVEF+Phase Bandwidth | 60 | M2 | 0.140 | 0.044 | 0.198 | 0.004 |
| TPD+LVEF+Phase Bandwidth | 60 | M3 | 0.009 | 0.000 | 0.023 | 0.036 |
| TPD | 72 | M1 | 0.023 | 0.008 | 0.045 | 0.004 |
| TPD | 72 | M2 | 0.143 | 0.041 | 0.257 | 0.000 |
| TPD | 72 | M3 | 0.037 | 0.000 | 0.052 | 0.000 |
| TPD+LVEF | 72 | M1 | 0.018 | 0.004 | 0.037 | 0.000 |
| TPD+LVEF | 72 | M2 | 0.186 | 0.095 | 0.271 | 0.000 |
| TPD+LVEF | 72 | M3 | 0.019 | 0.000 | 0.031 | 0.000 |
| TPD+LVEF+Phase SD | 72 | M1 | 0.018 | 0.003 | 0.034 | 0.008 |
| TPD+LVEF+Phase SD | 72 | M2 | 0.185 | 0.065 | 0.264 | 0.000 |
| TPD+LVEF+Phase SD | 72 | M3 | 0.017 | 0.000 | 0.035 | 0.000 |
| TPD+LVEF+Phase Bandwidth | 72 | M1 | 0.018 | 0.004 | 0.036 | 0.000 |
| TPD+LVEF+Phase Bandwidth | 72 | M2 | 0.186 | 0.077 | 0.274 | 0.004 |
| TPD+LVEF+Phase Bandwidth | 72 | M3 | 0.010 | 0.000 | 0.028 | 0.016 |

LVEF = left ventricular ejection fraction; TPD = total perfusion deficit.
