# Supplementary material for: Phase entropy of gated SPECT-MPI for predicting major adverse cardiovascular events: incremental prognostic value beyond perfusion and function
Source: Front Cardiovasc Med. 2026 Jul 7;13:1839622. doi: 10.3389/fcvm.2026.1839622 (PMC13385191; doi:10.3389/fcvm.2026.1839622)
Supplement: Supplementary file 2 [file Datasheet2.docx]

**Supplementary Table 2. Risk reclassification over time between the model 1 and model 3.**

| **Follow-up (months)** | **Group** | **Model 1 Risk Category** | **Model 3: Low <20%** | **Model 3:** **Intermediate 20–40%** | **Model 3:** **High ≥40%** | **Total** |
| --- | --- | --- | --- | --- | --- | --- |
| **12** | **All patients** | **Low <20%** | **1674** | **0** | **0** | **1674** |
|  |  | **Intermediate 20–40%** | **0** | **0** | **0** | **0** |
|  |  | **High ≥40%** | **0** | **0** | **0** | **0** |
|  | **Patients with MACE** | **Low <20%** | **207** | **0** | **0** | **207** |
|  |  | **Intermediate 20–40%** | **0** | **0** | **0** | **0** |
|  |  | **High ≥40%** | **0** | **0** | **0** | **0** |
|  | **Patients without MACE** | **Low <20%** | **1467** | **0** | **0** | **1467** |
|  |  | **Intermediate 20–40%** | **0** | **0** | **0** | **0** |
|  |  | **High ≥40%** | **0** | **0** | **0** | **0** |
| **24** | **All patients** | **Low <20%** | **488** | **104** | **0** | **592** |
|  |  | **Intermediate 20–40%** | **0** | **1082** | **0** | **1082** |
| **0** |  | **High ≥40%** | **0** | **0** | **0** | **0** |
|  | **Patients with MACE** | **Low <20%** | **89** | **19** | **0** | **108** |
|  |  | **Intermediate 20–40%** | **0** | **302** | **0** | **302** |
|  |  | **High ≥40%** | **0** | **0** | **0** | **0** |
|  | **Patients without MACE** | **Low <20%** | **367** | **79** | **0** | **446** |
|  |  | **Intermediate 20–40%** | **0** | **692** | **0** | **692** |
|  |  | **High ≥40%** | **0** | **0** | **0** | **0** |
| **36** | **All patients** | **Low <20%** | **0** | **0** | **0** | **0** |
|  |  | **Intermediate 20–40%** | **0** | **1307** | **367** | **1674** |
|  |  | **High ≥40%** | **0** | **0** | **0** | **0** |
|  | **Patients with MACE** | **Low <20%** | **0** | **0** | **0** | **0** |
|  |  | **Intermediate 20–40%** | **0** | **358** | **163** | **521** |
|  |  | **High ≥40%** | **0** | **0** | **0** | **0** |
|  | **Patients without MACE** | **Low <20%** | **0** | **0** | **0** | **0** |
|  |  | **Intermediate 20–40%** | **0** | **710** | **151** | **861** |
|  |  | **High ≥40%** | **0** | **0** | **0** | **0** |
| **48** | **All patients** | **Low <20%** | **0** | **0** | **0** | **0** |
|  |  | **Intermediate 20–40%** | **0** | **530** | **62** | **592** |
|  |  | **High ≥40%** | **0** | **254** | **828** | **1082** |
|  | **Patients with MACE** | **Low <20%** | **0** | **0** | **0** | **0** |
|  |  | **Intermediate 20–40%** | **0** | **156** | **17** | **173** |
|  |  | **High ≥40%** | **0** | **78** | **344** | **422** |
|  | **Patients without MACE** | **Low <20%** | **0** | **0** | **0** | **0** |
|  |  | **Intermediate 20–40%** | **0** | **227** | **28** | **255** |
|  |  | **High ≥40%** | **0** | **95** | **287** | **382** |
| **60** | **All patients** | **Low <20%** | **0** | **0** | **0** | **0** |
|  |  | **Intermediate 20–40%** | **0** | **488** | **104** | **592** |
|  |  | **High ≥40%** | **0** | **254** | **828** | **1082** |
|  | **Patients with MACE** | **Low <20%** | **0** | **0** | **0** | **0** |
|  |  | **Intermediate 20–40%** | **0** | **153** | **33** | **186** |
|  |  | **High ≥40%** | **0** | **83** | **364** | **447** |
|  | **Patients without MACE** | **Low <20%** | **0** | **0** | **0** | **0** |
|  |  | **Intermediate 20–40%** | **0** | **138** | **34** | **172** |
|  |  | **High ≥40%** | **0** | **56** | **184** | **240** |
| **72** | **All patients** | **Low <20%** | **0** | **0** | **0** | **0** |
|  |  | **Intermediate 20–40%** | **0** | **266** | **326** | **592** |
|  |  | **High ≥40%** | **0** | **0** | **1082** | **1082** |
|  | **Patients with MACE** | **Low <20%** | **0** | **0** | **0** | **0** |
|  |  | **Intermediate 20–40%** | **0** | **78** | **113** | **191** |
|  |  | **High ≥40%** | **0** | **0** | **461** | **461** |
|  | **Patients without MACE** | **Low <20%** | **0** | **0** | **0** | **0** |
|  |  | **Intermediate 20–40%** | **0** | **22** | **36** | **58** |
|  |  | **High ≥40%** | **0** | **0** | **76** | **76** |

Risk stratification was defined by cumulative MACE probability at each follow-up time point: Low risk < 20%, Intermediate risk 20–40%, High risk ≥ 40%. MACE = major adverse cardiac events; LVEF = left ventricular ejection fraction; TPD = total perfusion deficit.
